# *In Vitro* Amplification of Misfolded Prion Protein Using Lysate of Cultured Cells

**DOI:** 10.1371/journal.pone.0018047

**Published:** 2011-03-28

**Authors:** Charles E. Mays, Jihyun Yeom, Hae-Eun Kang, Jifeng Bian, Vadim Khaychuk, Younghwan Kim, Jason C. Bartz, Glenn C. Telling, Chongsuk Ryou

**Affiliations:** 1 Department of Microbiology, Immunology, and Molecular Genetics, University of Kentucky College of Medicine, Lexington, Kentucky, United States of America; 2 Sanders-Brown Center on Aging, University of Kentucky College of Medicine, Lexington, Kentucky, United States of America; 3 Department of Medical Microbiology and Immunology, Creighton University, Omaha, Nebraska, United States of America; Ohio State University, United States of America

## Abstract

Protein misfolding cyclic amplification (PMCA) recapitulates the prion protein (PrP) conversion process under cell-free conditions. PMCA was initially established with brain material and then with further simplified constituents such as partially purified and recombinant PrP. However, availability of brain material from some species or brain material from animals with certain mutations or polymorphisms within the PrP gene is often limited. Moreover, preparation of native PrP from mammalian cells and tissues, as well as recombinant PrP from bacterial cells, involves time-consuming purification steps. To establish a convenient and versatile PMCA procedure unrestricted to the availability of substrate sources, we attempted to conduct PMCA with the lysate of cells that express cellular PrP (PrP^C^). PrP^Sc^ was efficiently amplified with lysate of rabbit kidney epithelial RK13 cells stably transfected with the mouse or Syrian hamster PrP gene. Furthermore, PMCA was also successful with lysate of other established cell lines of neuronal or non-neuronal origins. Together with the data showing that the abundance of PrP^C^ in cell lysate was a critical factor to drive efficient PrP^Sc^ amplification, our results demonstrate that cell lysate in which PrP^C^ is present abundantly serves as an excellent substrate source for PMCA.

## Introduction

Conformational conversion of the α helix rich cellular prion protein (PrP^C^) to the β sheet rich scrapie prion protein (PrP^Sc^) is the major biochemical event that characterizes prion diseases [1]. The protein-only hypothesis postulates that prion replication is facilitated by PrP^Sc^ functioning as a template to convert PrP^C^ into the disease-associated conformation [2]. Although PrP conversion in cultured cells and animal models is possible, it has been quite difficult to reproduce the process *in vitro*. To establish an *in vitro* system that supports misfolding of PrP, a number of assays have been devised (reviewed in [3]).

Protein misfolding cyclic amplification (PMCA) is an assay that mimics the PrP^Sc^ propagation process under cell-free conditions. This method amplifies misfolded PrP by converting PrP^C^ to PrP^Sc^ during incubation with periodic sonication [4]. PrP^Sc^ generated by PMCA is infectious in wild-type animals [5] and can be indefinitely propagated with preserved properties of the original PrP^Sc^ [5]–[7]. PMCA recapitulates the species barrier of prion transmission [8]–[11], prion strain interference [7], and *de novo* generation of prions [12], [13]. Furthermore, PMCA is quite useful in studying the cofactors that influence PrP conversion [14]–[24], and in detecting PrP^Sc^ from biological samples of humans and animals [25]–[37].

PMCA has contributed to a number of important perspectives in prion biology, however, its conventional application to certain investigations still faces a few challenging problems. One of these problems is associated with the source of PMCA substrate. PMCA was originally designed to use brain homogenate derived from healthy animals that contains an excess amount of PrP^C^, to which a minute amount of prion-infected brain material, the source of PrP^Sc^, was diluted [4]. This prototypic method has evolved to use the lipid raft fractions of the plasma membrane as the source for PrP^C^ [23], [38], [39] because PrP conversion occurs at the caveolae-like membrane domains of neuronal cells [40]–[42]. Recently, PrP^C^ purified from brain tissue or cultured mammalian cells [19], [43] and recombinant PrP expressed in bacterial cells [30], [44], [45] have replaced brain material for PMCA. Crude brain homogenate and the lipid raft fractions of the membrane provide a comprehensive set of components required for PMCA including a cofactor, while purified PrP^C^ or recombinant PrP offers defined minimal substrates. However, availability of brain material from certain species or transgenic animals carrying the PrP gene with certain mutations and polymorphisms is often limited. Alternatively, preparation of the substrates by expression/purification of native PrP^C^ from animal tissues and cell lines, as well as recombinant PrP from bacterial cells, requires additional, laborious steps. Thus, it is necessary to establish a convenient alternative that overcomes aforementioned drawbacks of the current PMCA method.

In this study, we used cell lysate of cultured mammalian cell lines in PMCA reactions. Lysate of cultured cells has not been used as a substrate source for PMCA and it has been considered incapable of supporting PrP^Sc^ formation in PMCA unless complemented with brain homogenate that may include a cofactor for PrP conversion [6], [46]. Based on our recent observation that PrP^C^ abundance is critical for robust PrP^Sc^ propagation in PMCA [21], we performed PMCA with PrP-expressing cell lysates in which the level of PrP^C^ was equivalent to wild type brain material. Here, we show that PMCA replication of mouse and hamster-adapted PrP^Sc^ using cell lines that express murine and hamster PrP^C^, respectively.

## Results

### Estimation of the PrP^C^ level in cell lines

**Figure 1:**
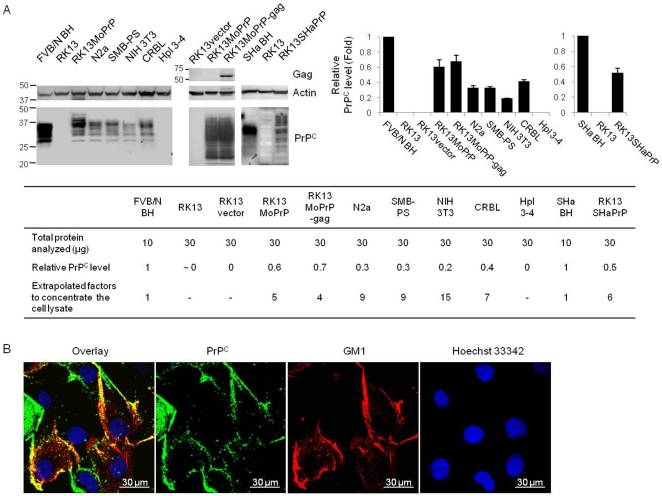
Expression of PrP^C^ in a variety of cell lines. (A) Comparison of the PrP^C^ levels in cell lines and brain homogenate. Western blotting followed by densitometry demonstrated relative differences of the PrP^C^ levels. Extrapolation of multiplication factors to concentrate cell lysate was based on the amount of protein analyzed and the relative PrP^C^ levels. PrP^C^ was detected by D13 (left blot), 6H4 (middle blot), and 3F4 (right blot) antibodies. (B) Fluorescence images of RK13 cells expressing full length mouse PrP^C^. Colocalization (yellow, overlay) of PrP^C^ (green) and GM1 (red) in the lipid rafts of the plasma membrane of RK13MoPrP was shown by confocal microscopy. The nuclei (blue) were stained by Hoechst 33258. Scale was shown by a 30 µm bar.

We established RK13 cells that express the full-length mouse and Syrian hamster PrP open reading frames, designated RK13MoPrP and RK13SHaPrP. We compared the level of PrP^C^ in RK13MoPrP to that of FVB/N wild type brain homogenate and several cell lines: N2a, SMB-PS, NIH 3T3, CRBL, and Hpl-3-4 cells (Fig. 1A). Western blot analysis indicated that RK13MoPrP cells expressed PrP^C^ at a higher level than other PrP^C^-expressing cell lines. However, the abundance of PrP^C^ in RK13MoPrP cells was lower than wild type mouse brain, but similar to RK13MoPrP-gag cells in which the retroviral gag gene was coexpressed with mouse PrP. Among previously established cell lines, the level of PrP^C^ in N2a, SMB-PS, and CRBL was similar to each other, but greater compared to NIH 3T3. RK13, RK13vector, and Hpl 3–4 lacked PrP^C^ expression. RK13SHaPrP cells expressed PrP^C^ at a lower level than wild type Syrian hamster brain.

Next, we investigated whether PrP^C^ expressed in our newly established RK13 cell line colocalized to lipid rafts in the plasma membrane as observed in nature. Immunofluorescence microscopy revealed that the location of PrP^C^ expression in RK13MoPrP cells was at the cell surface and colocalized with the glycosphingolipid GM1, a marker for lipid rafts (Fig. 1B). This suggests that PrP^C^ expressed in RK13MoPrP cells was processed normally and localized as usually found in nature.

### PMCA propagation of PrP^Sc^ is supported using RK13MoPrP and RK13MoPrP-gag cell lysate

**Figure 2:**
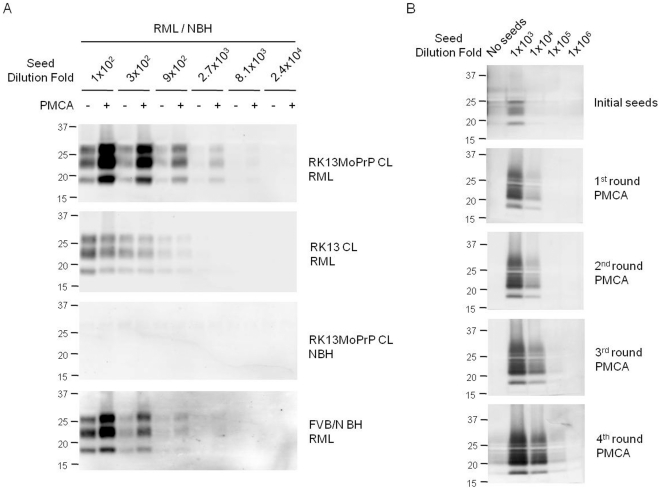
PMCA using cell lysate of RK13MoPrP and RK13. (A) Amplification of protease-resistant PrP^Sc^ of RML prions. The level of PK-resistant PrP^Sc^ before (−) and after (+) PMCA was compared by Western blotting using monoclonal anti-PrP 6H4 antibody. Prion-sick (RML) and normal (NBH) brain homogenate were diluted 100–24,000 fold in cell lysate (CL) of RK13MoPrP and RK13. PMCA using wild type FVB/N brain homogenate (BH) was conducted as a control. (B). Serial PMCA with RK13MoPrP cell lysate as substrates. Initially, RML seeds were diluted 100–1,000,000 fold, and then the products of PMCA in each round were diluted 10 fold thereafter. The PK-resistant PMCA products were detected by Western blotting using monoclonal anti-PrP 6H4 (panel A) and 5C6 (panel B) antibodies.

RK13MoPrP cell lysate was applied to PMCA for PrP^Sc^ amplification. Similar levels of PrP^C^ from RK13MoPrP cells and murine brain were used in PMCA (Fig. 1A). The PrP^C^ in RK13MoPrP cell lysate was converted into PrP^Sc^ when seeded with RML prions (Fig. 2A, top panel). This result was similar to PMCA using wild type brain homogenate (Fig. 2A, bottom panel). As expected, PrP^Sc^ of RML (Rocky Mountain Laboratory) prions was not propagated in the absence of PrP^C^ when lysate of untranfected RK13 cells was used in PMCA (Fig. 2A, second panel). Normal brain homogenate did not induce spontaneous PrP^Sc^ formation from RK13MoPrP cell lysate (Fig. 2A, third panel). In serial PMCA, RK13MoPrP cell lysate was able to support continuous propagation of PrP^Sc^ (Fig. 2B).

**Figure 3:**
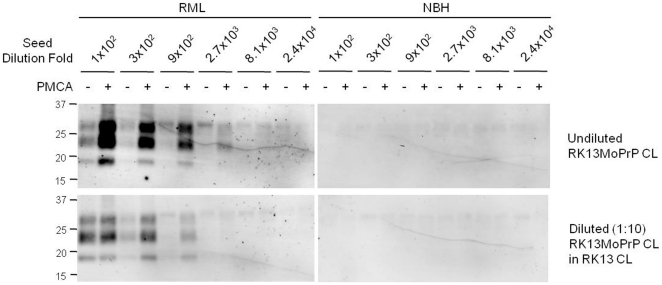
PrP^Sc^ amplification affected by PrP^C^ abundance in cell lysate. PMCA was performed using undiluted and diluted (1∶10 fold) RK13MoPrP cell lysate (CL). Both RML and NBH were used as seeds in dilutions of 100–24,000 fold. PK-treated pre- (−) and post-PMCA (+) samples were analyzed. Western blotting was performed using monoclonal anti-PrP 6H4 antibody.

To investigate if PrP^C^ abundance in cell lysate influences PMCA conversion of PrP^C^, we conducted PMCA with RK13MoPrP cell lysate diluted 1:10 in lysate of normal RK13 cells that do not express PrP^C^. Consistent with our previous PMCA studies with brain material [21], the amount of PrP^C^ present in cell lysate dictated the level of PrP^Sc^ generation in PMCA. PrP^Sc^ amplification with diluted RK13MoPrP cell lysate was less efficient than amplification with undiluted substrate (Fig. 3). Spontaneous generation of PrP^Sc^ in PMCA was not observed when normal brain material was used as the seeding source (Fig. 3).

**Figure 4:**
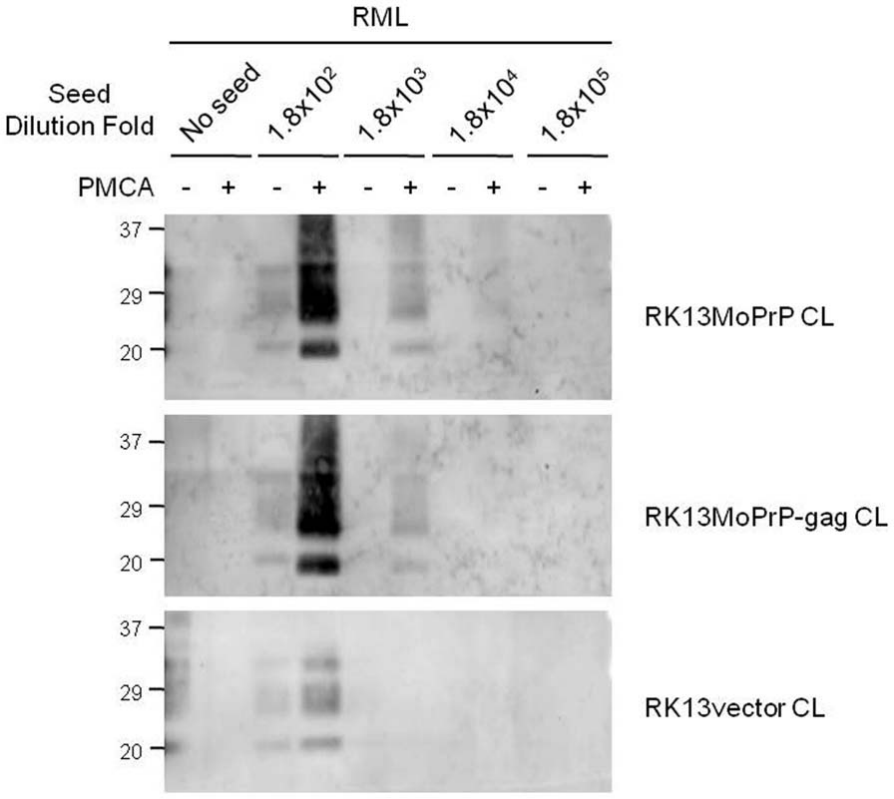
Influence of Gag expression to PrP^Sc^ amplification. PMCA using RK13MoPrP and RK13MoPrP-gag cell lysate was conducted with (1,800–180,000 fold diluted) or without (no seeds) RML seeds. Cell lysate of RK13vector that lacks expression of both PrP^C^ and Gag was used as a control. Pre- (−) and post-PMCA (+) samples were treated with PK and analyzed by and monoclonal anti-PrP 5C6 antibody was used for Western blotting.

Because expression of the human immunodeficiency virus-1 (HIV-1) gag gene in cultured cells promotes formation of PrP^Sc^ in the cell culture models of prion disease [47], we assessed whether HIV-1 Gag influences *in vitro* amplification of PrP^Sc^. To address this, we performed PMCA using RK13MoPrP-gag cell lysate as a substrate and PrP^Sc^ from RML prions as seeds. We failed to detect a significant difference in PrP^Sc^ conversion efficiency using PMCA between RK13MoPrP-gag and RK13MoPrP cell lysates (Fig. 4), suggesting that HIV-1 Gag does not affect PrP^Sc^ conversion.

### Lysate of both neuronal and non-neuronal cell lines supports PMCA generation of PrP^Sc^

**Figure 5:**
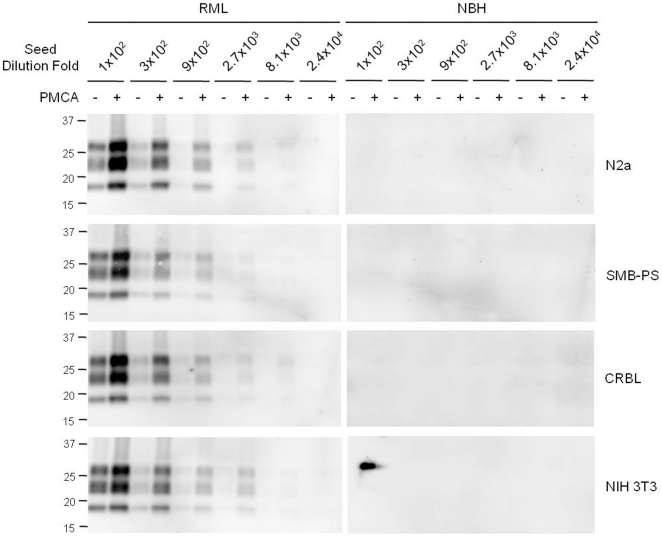
PMCA using lysates of a wide range of cell types. Cell lysate of neuronal (N2a), prion-free brain mesenchymal (SMB-PS), mixed cerebellar neuronal and glial (CRBL) or fibroblast (NIH 3T3) cells was concentrated to include the PrP^C^ level of wild brain homogenate. PMCA was performed by seeding with RML-sick (RML) and normal (NBH) brain homogenate. The seed dilution fold was 100–24,000. The PrP^Sc^ level of PMCA before (−) and after (+) was compared. Monoclonal anti-PrP 6H4 antibody was used for Western blotting.

To assess the ability of lysate derived from previously established cell lines to support PMCA, we prepared cell lysate in which PrP^C^ was concentrated to the same level as wild type mouse brain material (Fig. 1A). The cell lines from diverse origins were chosen: N2a neuroblastoma cells exhibit characteristics of neurons [48]; SMB-PS cells derived from scrapie-infected mouse brain but cured by *in vitro* treatment with pentosan sulfate are originated from the mesenchymal lineage [49]; CRBL cells derived from the cerebellum of p53 null mice show expression of both glial and neuronal markers [50]; and NIH 3T3 cells feature the characteristics of common fibroblasts [51]. All of the tested lysates supported PrP^Sc^ propagation in PMCA (Fig. 5), suggesting that PrP conversion is not exclusively dependent on the neuronal cells.

### The efficiency of PMCA formation of PrP^Sc^ varies upon the cellular source of PrP^C^

**Figure 6:**
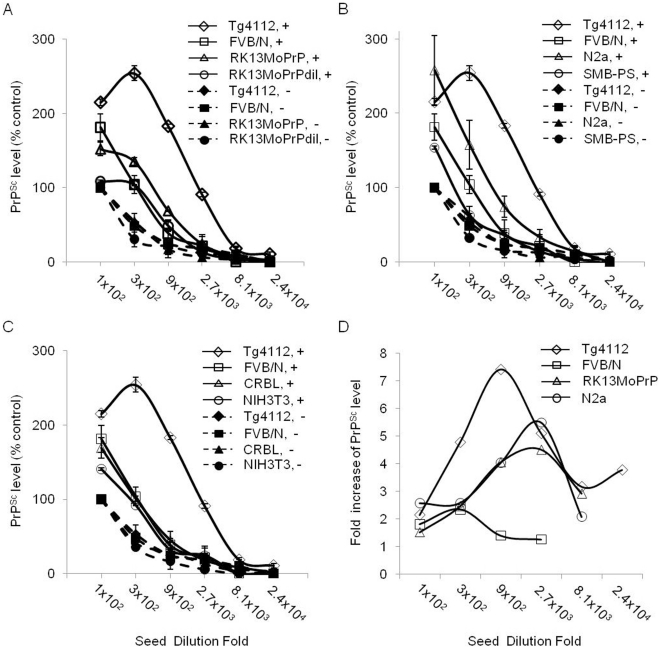
Comparison of ability of cell lysate to support PMCA. (A–C) The levels of PrP^Sc^ in pre- (−, dashed line with filled symbols) and post-PMCA (+, solid line with open symbols) samples shown in Figures 2, 3, and 4 were measured by densitometry and presented as relative % to that of the 1∶100 diluted PrP^Sc^ seeds (n = 3 each). (D) Efficacy of PMCA supported by different PrP^C^ sources was presented as fold increase of the PrP^Sc^ level compared to the PrP^Sc^ level of seeds in each dilution. The data sets for transgenic mice that overexpress PrP^C^ in the brain (Tg4112) was obtained from the Western blots previously published [21].

We compared the efficiency of PMCA generation of PrP^Sc^ using cell lysate from different sources. Based on the densitometric analysis of the Western blots, we plotted the normalized PrP^Sc^ levels of each pre- and post-PMCA sample. The PrP^Sc^ levels amplified with RK13MoPrP and N2a cell lysate were greater than those generated by wild type brain homogenate, but less than those generated by Tg(MoPrP)4112 [21] brain homogenate (Fig. 6A and B). The PrP^Sc^ levels amplified with CRBL and NIH3T3 cell lysate were almost identical with those generated by wild type brain homogenate (Fig. 6C). The SMB-PS cell lysate was not obviously better than wild type brain homogenate or cell lysate of other cell types (Fig. 6B). To further investigate the efficacy of RK13MoPrP and N2a cell lysate in generating PrP^Sc^, the fold increase of newly synthesized PrP^Sc^ was plotted. The rates of PrP^Sc^ generation by RK13MoPrP and N2a cell lysate were intermediate between those obtained with Tg4112 and wild type brain homogenate (Fig. 6D). Similar analysis for cell lysate of other cell lines indicated that the rate of PrP^Sc^ formation resembled that of wild type brain homogenate (data not shown).

### PMCA propagation of PrP^Sc^ is supported using lysate of RK13 cells that express Syrian hamster PrP^C^

**Figure 7:**
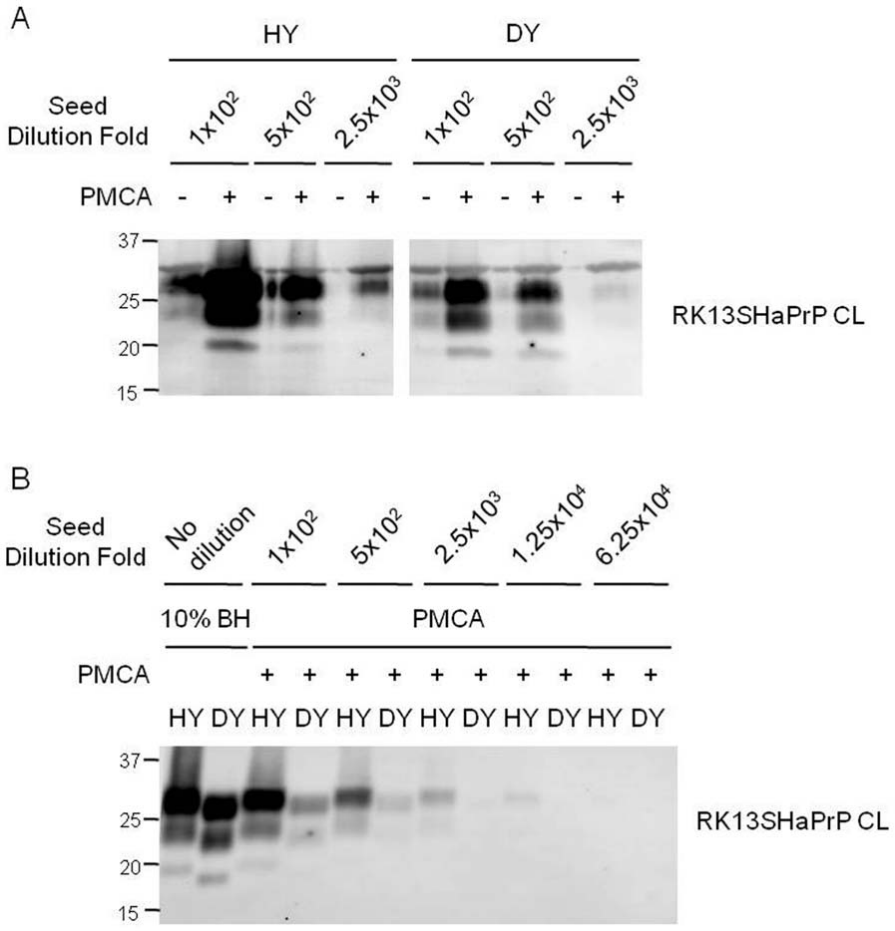
PMCA using cell lysate of RK13SHaPrP. (A) PK-resistant PrP^Sc^ amplification of two Syrian hamster-adapted TME prions (HY and DY) with cell lysate (CL) of RK13SHaPrP. The HY and DY seeds were diluted 100–2,500 fold for PMCA. The level of PrP^Sc^ in pre- (−) and post-PMCA (+) samples was analyzed by Western blotting. (B) Comparison of the PK-resistant PrP^Sc^ of HY and DY prions generated by PMCA. The HY and DY seeds were diluted 100–62,500 fold for PMCA. Ten % brain homogenate (BH) of HY- and DY-sick Syrian hamsters were used as controls. PrP^Sc^ in both panels A and B was detected by monoclonal anti-PrP 3F4 antibody.

To address that cell lysate-based PMCA is not restricted to murine PrP^C^ and murine-adapted scrapie prions, we performed PMCA using Syrian hamster PrP^C^ and the hyper (HY) and drowsy (DY) strains of hamster-adapted transmissible mink encephalopathy (TME) prions [52]. PrP^Sc^ from either HY or DY strains was successfully amplified in PMCA using RK13SHaPrP cell lysate (Fig. 7A). The strain-specific migration of HY and DY PrP^Sc^ was similar between brain-derived and PMCA-generated material (Fig. 7B). In particular, HY was more efficiently amplified than DY in cell lysate-based PMCA (Fig. 7B), which corresponds to the results of PMCA studies that used Syrian hamster brain homogenate and bioassays of HY and DY in Syrian hamsters [7].

## Discussion

The current study demonstrates that cell lysate with concentrated PrP^C^ allowed robust PMCA of PrP^Sc^ from multiple strains and species. The ability of cell lysate to support PrP^Sc^ formation in PMCA is comparable to that of wild type brain material. This result suggests that cell lysate can replace animal organ-derived material for *in vitro* PrP^Sc^ amplification without compromising PrP conversion efficiency.

Previously, cultured cell lysate was shown to support extremely low levels of PrP^Sc^ formation [46]. Moreover, PMCA with lysate of animal cells has been reported to require compensation with PrP^−/−^ brain material for adequate amplification [6], suggesting that additional factors derived from the brain material are required to properly amplify PrP^Sc^ using cell lysate. In contrast to these results, our study demonstrated that cell lysate is sufficient to support PrP^Sc^ amplification, although inclusion of the “auxiliary factors” in cell lysate remains to be elucidated. The discrepancy between previous and current results may be due to the abundance of PrP^C^ in PMCA. As opposed to the previous studies using cell lysate with the endogenous level of PrP^C^, our study exploited cell lysate with concentrated PrP^C^. As demonstrated in our previous [21] and current studies, the abundance of PrP^C^ is a critical factors to guarantee robust PrP^Sc^ amplification.

Our data suggest that, by adjusting PrP^C^ levels to wild type brain material, lysate of newly engineered and previously established animal cell lines can serve as an excellent substrate for PMCA, in which PrP^C^ maintains its native states of subcellular localization, conformation, glycosylation, disulfide bridging, and glycosylphosphatidylinositol anchoring [40], [42], [53], [54]. Since these post-translational modification states can affect the efficiency of PMCA [17], [22], use of material that include PrP^C^ in its native form localized in the lipid rafts might result in more efficient PMCA than a similar reaction with recombinant PrP that lacks native conformation and post-translational modifications.

Interestingly, our results demonstrated that HIV-1 Gag does not affect PrP^Sc^ conversion, suggesting that retroviral Gag functions in PrP^Sc^ propagation through the mechanism other than the PrP conversion process. Instead, it may play a role in the regulatory mechanisms for prion susceptibility and maintenance of prion infection at the cellular level as evidenced by Gag-enhanced release of mouse-adapted scrapie from cell cultures [55].

To date, the existence of the “auxiliary factors” for PrP conversion remains unknown. Recent PMCA studies showed that conversion of purified and recombinant PrP^C^ to infective PrP^Sc^ was successful in the absence of any cofactor [44]. In contrast, other lines of PMCA-based investigations suggest the presence of a cofactor for PrP conversion [14], [23], [24], [45], [56]. Some demonstrate enhanced PrP conversion by supplementation with undefined crude homogenate or partially purified fractions of cells and tissues [23], [39], [56], while others specify RNA [14] and plasminogen [24] for the cofactor activity. However, the tissue specific presence or utilization of this cofactor remains to be further investigated. A recent report proposed the existence of a brain-specific cofactor [57], whereas other studies indicated the presence of the cofactor is not limited to the brain. In fact, PrP conversion by PMCA is successful with spleen and muscle tissue homogenate [58] and PMCA with purified PrP^C^ is equally robust when supplemented with tissue homogenate of major wild type organs including the brain [23]. In agreement, our results showed that lysate of both neuronal and non-neuronal cells supported robust PMCA independent of the origin of the cell types. This suggests that the cofactor for PrP conversion is unlikely to be present exclusively in the brain or in neurons, if it exists.

Cell lysate-based PMCA may provide convenience in examining PrP conversion influenced by the PrP^C^ sequence variability. PrP^C^ of various species or with either mutations or polymorphisms can be expressed in the mammalian cell lines and the cell lysate can be used for PMCA without purification. This offers a practical alternative to using brain material of limited availability or purified and recombinant PrP that require laborious, time-consuming procedures for preparation.

In summary, lysate of cultured cells that express PrP^C^ is an excellent substrate source to amplify PrP^Sc^ in PMCA. Validation of cell lysate-based PMCA introduces a convenient model system that provides unlimited flexibility in functional analysis of PrP conversion associated with diverse PrP sequence variances.

## Materials and Methods

### Ethics statement

The experiments using animals were carried out in strict accordance with the recommendations in the Guide for the Care and Use of Laboratory Animals of the National Institutes of Health. The protocol was approved by the Institutional Animal Care and Use Committee the University of Kentucky (IACUC ID Number: 2006-0044). The procedures to establish new cell lines were carried out based on the protocol approved by the Institutional Biosafety Committee of the University of Kentucky (Registration Number: B10-795).

### Cell lines

The cell lines used for the study are following: a rabbit kidney epithelial cell line RK13(ATCC, CCL37) [59]; a mouse neuroblastoma cell line, N2a (ATCC, CCL131) [48]; a scrapie-infected mouse brain cell line cured by pentosan sulfate, SMB-PS [49]; a mouse fibroblast cell line, NIH3T3 (ATCC, CRL1658) [51]; a mouse cerebellar cell line, CRBL [50]; a PrP knockout mouse hippocampal neuronal cell line, Hpl 3–4 [60]; two RK13 cell lines that either express mouse PrP^C^ alone (RK13MoPrP) or coexpress both mouse PrP^C^ and retroviral Gag protein (RK13MoPrP-gag); and RK13 cells that express Syrian hamster PrP^C^ (RK13SHaPrP). RK13MoPrP and RK13SHaPrP were established by stable transfection of RK13 cells that lack expression of endogenous PrP^C^ with the PrP open reading frames of each species cloned in the mammalian expression vector pIRESpuro3 (Clonetech, PaloAlto, CA) by the method described previously [61]. RK13vector was established by transfection with empty pIRESpuro3 plasmid. The stable transfectants were obtained by selection with 1 µg/ml puromycin. RK13MoPrP-gag was established by stably transfecting RK13MoPrP with pcDNA3-gag that harbors the gene for HIV-1 Gag precursor protein. RK13MoPrP-gag was selected with 1 mg/ml Geneticin sulfate (G418, Invitrogen, Carlsbad, CA), as previously described [47].

### Cell culture

Culture of the cell lines were conducted as described elsewhere [50]. Briefly, cells were grown in Dulbecco's Modified Eagle's Medium (high glucose, Invitrogen) containing 10% fetal bovine serum, 1% penicillin-streptomycin, 1% glutamax under saturated humidity and 5% CO_2_ conditions at 37°C.

### Fluorescence microscopy

RK13MoPrP cells cultured onto 22×22 mm square glass cover slips were fixed with 4% paraformaldehyde and blocked with 10% goat serum (Invitrogen). For PrP staining, cells were incubated with 0.5 µg/ml monoclonal anti-PrP primary antibody, 6H4 (1∶500 dilution) (Prionics, Zurich, Switzerland) and fluorescein-conjugated goat anti-mouse IgG secondary antibody (1∶500 dilution) (Jackson ImmunoResearch Lab Inc, West Grove, PA) for 45 min each at room temperature. For GM1 staining, cell were incubated with the Alexa Fluor 594-conjugated cholera toxin B subunit (1∶50 dilution) (Invitrogen)for 45 min. Counterstaining of nuclei using 5 µg/ml Hoechst 33342 dye (Invitrogen) was performed simultaneously during incubation with the secondary antibody. The cover slips mounted on glass slides with 15 µl Mowiol mounting medium were immediately observed via a Leica AOBS TCS SP5 inverted laser scanning confocal microscope.

### PMCA with cell lysate

PMCA was conducted by using cell derived material as the PrP^C^ source. Cultured cells in the 100 mm-diameter culture plates were washed twice with 10 ml phosphate buffered saline (PBS) The adhered cells were harvested in 1ml PBS using a cell scraper. Depending on the level of PrP^C^ in each cell line, cells in 5–15 plates were combined and centrifuged at 1,000×*g* for 5 min at 4°C. Pellet was washed with 10ml PBS once again before resuspended in 1 ml PMCA conversion buffer [PBS, pH 7.2, 150 mM NaCl, 1% Trition X-100, 4 mM EDTA, 1X CompleteMini protease inhibitors (Roche)]. Cell lysate was made by serially passaging through hypodermic needles from 16 to 21 gauges. After centrifugation at 2,000×*g* for 5 min at 4°C, supernatant was used as the substrate for PMCA.

Brain homogenate (10% w/v) of terminally ill FVB mice inoculated with RML prions or Syrian hamsters inoculated with either the HY or DY TME agents was used as the source for PrP^Sc^ seeds. For PMCA, the seed was diluted as indicated (10^2^–10^6^ fold) in 0.1 ml cell lysate. Then, PMCA was performed as previously described [21]. Briefly, 94 cycles of amplification was conducted at 37°C with periodic 40 s sonication every 30 min using Misonix Model 3000 (Farmingdale, NY). For serial PMCA, the PMCA products from previous amplification rounds were diluted 10 fold in 0.1 ml of fresh cell lysate and subjected to repeated cycles of amplification as described above. Total four rounds of PMCA were carried out for the current study.

### Western blot analysis

Western blotting for cell lysate to measure the expression level of PrP^C^ was performed with samples not treated with PK. However, the pre- and post-PMCA samples were treated with PK (100 µg/ml) for 1 hr at 37°C, and then subjected to Western blotting. The procedure followed the protocol described in the previous publication [50]. Mouse and Syrian hamster PrP was recognized by incubation with monoclonal anti-PrP antibodies, 6H4 (Prionics, Zurich, Switzerland), D13 (Inpro, South San Francisco, CA), 3F4 (Signet Laboratory, Boston, MA), and 5C6 (raised against full length recombinant cervid PrP. G. Telling, unpublished data). Monoclonal anti-β-actin (ACTN05, Neomarker, Fremont, CA) and anti-HIV p24 (Chemicon, Temecula, CA) antibodies were used to detect the expression of β-actin and Gag, respectively. The peroxidase conjugated goat anti-mouse IgG (Pierce, Rockford, IL) antibody was used as the secondary antibody. The signal was developed by using the ECL plus substrate (Amersham Pharmacia, Piscataway, NJ) and visualized by scanning with the Fuji Film FLA 5000 image reader (Fuji Film, Edison, NJ).

Doc-It Image Analysis Software (UVP, Upland, CA) was used for densitometry analysis. Densitometry data were used to estimate the levels of PrP^C^ expressed in each cell line and PrP^Sc^ generated by PMCA. The level of newly generated PrP^Sc^ and efficiency of PMCA was calculated as described previously [21].
